# Ecology and Caudal Skeletal Morphology in Birds: The Convergent Evolution of Pygostyle Shape in Underwater Foraging Taxa

**DOI:** 10.1371/journal.pone.0089737

**Published:** 2014-02-26

**Authors:** Ryan N. Felice, Patrick M. O’Connor

**Affiliations:** 1 Department of Biological Sciences, Ohio University, Athens, Ohio, United States of America; 2 Ohio Center for Ecology and Evolutionary Studies, Ohio University, Athens, Ohio, United States of America; 3 Department of Biomedical Sciences, Ohio University Heritage College of Osteopathic Medicine, Athens, Ohio, United States of America; University of Pennsylvania, United States of America

## Abstract

Birds exhibit a specialized tail that serves as an integral part of the flight apparatus, supplementing the role of the wings in facilitating high performance aerial locomotion. The evolution of this function for the tail contributed to the diversification of birds by allowing them to utilize a wider range of flight behaviors and thus exploit a greater range of ecological niches. The shape of the wings and the tail feathers influence the aerodynamic properties of a bird. Accordingly, taxa that habitually utilize different flight behaviors are characterized by different flight apparatus morphologies. This study explores whether differences in flight behavior are also associated with variation in caudal vertebra and pygostyle morphology. Details of the tail skeleton were characterized in 51 Aequornithes and Charadriiformes species. Free caudal vertebral morphology was measured using linear metrics. Variation in pygostyle morphology was characterized using Elliptical Fourier Analysis, a geometric morphometric method for the analysis of outline shapes. Each taxon was categorized based on flight style (flap, flap-glide, dynamic soar, etc.) and foraging style (aerial, terrestrial, plunge dive, etc.). Phylogenetic MANOVAs and Flexible Discriminant Analyses were used to test whether caudal skeletal morphology can be used to predict flight behavior. Foraging style groups differ significantly in pygostyle shape, and pygostyle shape predicts foraging style with less than 4% misclassification error. Four distinct lineages of underwater foraging birds exhibit an elongate, straight pygostyle, whereas aerial and terrestrial birds are characterized by a short, dorsally deflected pygostyle. Convergent evolution of a common pygostyle phenotype in diving birds suggests that this morphology is related to the mechanical demands of using the tail as a rudder during underwater foraging. Thus, distinct locomotor behaviors influence not only feather attributes but also the underlying caudal skeleton, reinforcing the importance of the entire caudal locomotor module in avian ecological diversification.

## Introduction

Understanding the processes that generate phenotypic diversity is an important goal in evolutionary biology [1], [2]. The evolutionary diversification of phenotypes can be influenced by many factors, including natural selection, sexual selection, biomechanical constraints, developmental processes, and trait interactions [2]–[8]. By testing hypotheses regarding the patterns and causes of morphological diversity in highly variable structures, we may better characterize the role that such variation has played in the diversification of clades [9]–[15].

**Figure 1:**
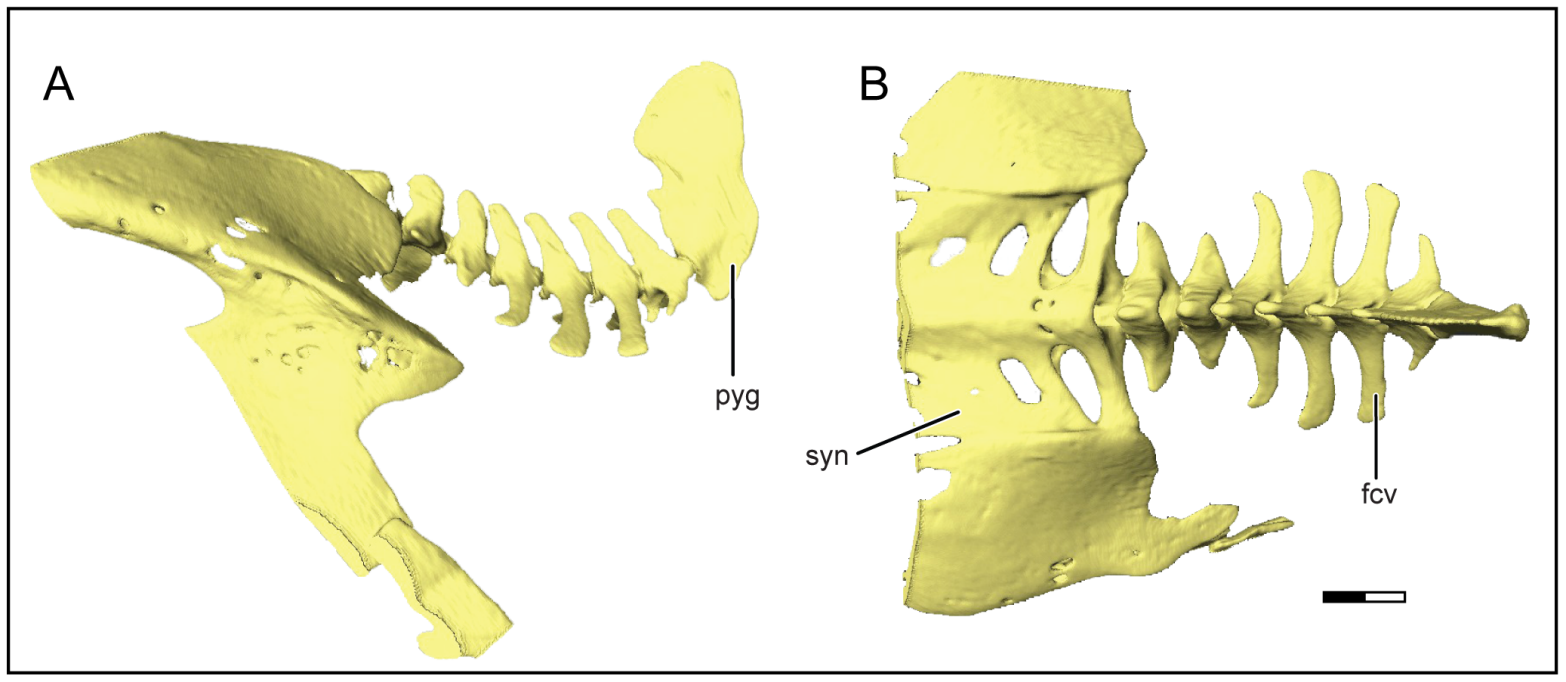
Avian caudal skeleton in left lateral (A) and dorsal (B) views. Abbreviations: fcv, free caudal vertebra; pyg, pygostyle; syn, synsacrum. Scale bar equals 5 mm.

The avian tail is one such highly variable structure, with modern birds using the tail as an integral component of the flight apparatus [16]–[18]. The role of the tail in flight is to supplement the lift produced by the wings during slow flight, reduce whole-body drag, and both stabilize and maneuver the bird during flight [19]–[22]. Bird tail morphology is specialized for its function as part of the locomotor apparatus and consists of an articulated fan of tail feathers, separate muscular systems for tail movements and tail fanning, and a modified, shortened tail skeleton [18]. The avian caudal skeleton consists of several (five to nine) free caudal vertebrae (Fig. 1). The terminal element of the caudal skeleton is the pygostyle, represented by a single, co-ossified unit consisting of the fused caudal-most vertebrae, ranging from three to seven in number [18], [23]. This serves as an attachment site for caudal musculature, tail feathers, and as an anchor for the tail fanning mechanism itself [16], [18].

The drivers of tail feather (rectrix: plural, rectrices) diversity are somewhat well understood. Tail fan shape determines the functional and aerodynamic properties of the tail [24], [25]. Not surprisingly then, tail-fan shape diversity reflects differences in ecology. As examples, birds that live in dense woodland environments benefit from the increased maneuverability granted by a long tail fan [22], whereas those that capture their prey in the air generally exhibit a deeply forked tail that increases agility [22]. High-speed fliers often have a shortened tail fan that reduces drag and thus increases flight efficiency, similar to the situation observed in long distance migrants [22], [26].

Tail-fan shape also serves non-aerodynamic functions. In some species, males exhibit an elaborate tail fan that deviates from the “optimal” shape predicted from aerodynamic models [27]–[31]. For example, male red-collared widowbirds in breeding plumage have a tail five times longer than that of females [32]. Sexually dimorphic rectrices like these are honest indicators of male quality and have been shown to evolve as a result of female preference [32], [33]. Such ornaments have evolved in numerous clades despite being energetically and aerodynamically costly [27], [32]. Thus, tail-fan phenotypic diversity is shaped not only by natural selection for increased flight performance, but also by sexual selection [27].

**Figure 2:**
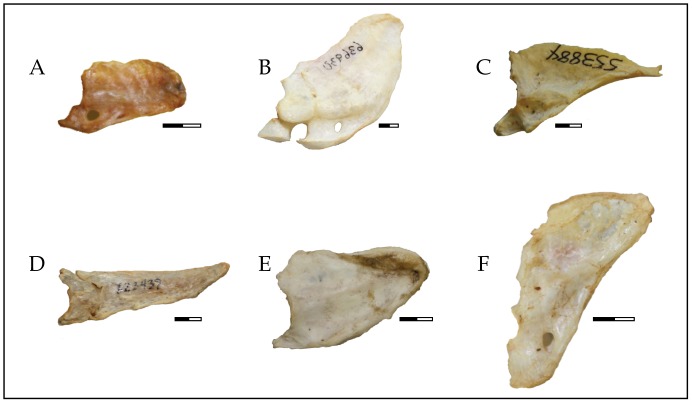
Pygostyle diversity. (A) Northern Fulmar (*Fulmaris glacialis* – AMNH 20697), (B) American White Pelican (*Pelecanus erythrorhynchos* – USNM 535930), (C) Great Cormorant (*Phalacrocorax carbo* – USNM 553884), (D) Adélie Penguin (*Pygoscelis adeliae* – AMNH 623439), (E) Common Loon (*Gavia immer* – FMNH 444970), and (F) Great Frigatebird (*Fregata minor* – FMNH 339432). Scale bar equals 5 mm.

In contrast to rectricial diversity, drivers of caudal skeletal diversity are poorly understood. The degree of morphological variation of the free caudal vertebrae and the pygostyle has long been recognized [34]–[39]. There is substantial interspecific variation in the number and form of the free caudal vertebrae in addition to the shape of the pygostyle (Fig. 2). However, little comparative consideration of caudal skeletal structure and function has been undertaken, with the exception of a few clades with highly specialized tails. For example, falconids and some hummingbirds have paired accessory pygostyle elements just ventral to the pygostyle. Accessory pygostyle bones are associated with the depressor caudae musculature [39]. These structures are hypothesized to have evolved to accommodate stresses on the tail during rapid maneuvering and braking in these highly aerial clades [39]. Woodpeckers (Picidae) are also noted for their derived caudal skeletal structure and function. Extremely arboreal woodpeckers use the tail as a prop for support during vertical climbing. The pygostyle of woodpeckers has a laterally expanded ventral surface (discus pygostyli) that increases the surface area for the attachment of both the rectrices and caudal musculature. The discus pygostyli is more expanded in species that use the tail as a prop more frequently (e.g., species that spend a considerable proportion of time utilizing the vertical or near-vertical components of the arboreal environment) and, as such, this derived caudal skeletal morphology has been interpreted as an adaptation for the unique function of the tail in woodpeckers [38], [40], [41].

The specialized caudal skeletal morphology in Falconidae and Picidae suggests that variation in caudal skeletal anatomy, like rectricial anatomy, likely evolves in response to variation in tail function. To date, there has been no broad comparative investigation of structure-function relationships in the avian caudal skeleton. Variation in forelimb (wing) skeletal morphology is strongly linked to flight style [42], [43]. For example, in Pelecaniformes, species that utilize different flight styles (e.g., flap, flap-glide, dynamic soar, static soar) are characterized by different forelimb skeletal anatomy. The length and diameter of the carpometacarpus (the forelimb element that supports the primary flight feathers of the wing) vary among functional groups, reflecting the different biomechanical demands of each flight style [43]. Likewise, hind limb morphology reflects aspects of ecology and locomotor behavior. For example, foot-propelled diving birds exhibit an enlarged area of attachment (i.e., the cnemial crest) for knee extensor musculature that functions to both stabilize the knee joint and produce powerful knee extension during swimming [44]. More generally, hind limb proportions can be used to discriminate among habitat types (e.g., arboreal, wading, swimming, terrestrial) with some confidence, suggesting that pelvic limb variation is influenced by differences in the locomotor demands of each substrate type [45]. The present study investigates the degree to which the avian caudal skeleton, like components of the appendicular skeleton, reflects differences in locomotor behavior.

Given that forelimb and hind limb skeletal anatomy differs among functional groups within birds, does caudal skeletal anatomy also exhibit clear structure-function relationships? As a framing statement then, we predict that birds that utilize different foraging strategies (e.g., aerial, terrestrial, pursuit diving) or flight styles (e.g., flap, soar, flap-glide) are characterized by variable caudal skeletal morphology that reflects this function. We examine this working hypothesis in a phylogenetic comparative context using morphometric data derived from both the free caudal vertebrae and the pygostyle and assess their relationships with both foraging and locomotor characteristics.

## Materials and Methods

### Taxon Sampling

Morphometric data were collected from 158 specimens representing a total of 51 species (35 genera, see Supplementary Table S1). Taxa were sampled primarily from the diverse waterbird assemblage, often referred to as the “Aequornithes” [46], [47]. Waterbirds include Ciconiiformes (storks), Gaviiformes (loons), Pelecaniformes (pelicans, cormorants, and allies), Procellariiformes (albatrosses and petrels), and Sphenisciformes (penguins) [46], [47]. Although the monophyly of this clade has been contested [48], [49], it was chosen as a focal group for several reasons. First, Aequornithes is among the most diverse avian groups in terms of morphology and body size range [47], [50]. Second, diversity in flight behavior and foraging behavior within this clade is well categorized [51]–[57]. Finally, taxa were sampled primarily from the waterbird assemblage because unlike many other neornithine clades, there is minimal-to-no sexual dimorphism of the tail feathers (rectrices) within the group [58]. Even taxa with elaborate rectrices, such as tropicbirds, are sexually monomorphic for this trait [59]. As such, differences in caudal skeletal morphology between males and females are not expected to influence the analyses conducted herein. In addition, six of the 51 taxa are not waterbirds but members of the somewhat distantly related Charadriiformes (shorebirds: *Larus argentatus, Stercorarius parasiticus, Fratercula cirrhata, Uria aalge, Cepphus columba, Ibidorhyncha struthersii*). These taxa are ecologically convergent with some waterbirds (e.g., alcids and penguins are both marine wing-propelled divers) and thus represent a useful comparison to waterbirds for understanding the correlated evolution of form and function.

In order to explore the relationship between caudal skeletal morphology and flight behavior, each taxon was assigned to both a flight style group and a foraging style group. These categorizations are based on published observations and other comparative ecomorphology studies [14], [43], [52], [55], [60]–[66]. The flight style categories were chosen as Flap, Flap-Glide, Dynamic Soar, Static Soar, Wing-Propelled Flightless and Foot-Propelled Flightless. Taxa were placed in one of five foraging style groups: Aerial, Terrestrial, Plunge Dive, Foot-Propelled Pursuit Dive, and Wing-Propelled Pursuit Dive. The aerial foraging group contains any taxon that habitually utilizes airborne foraging techniques including hawking, dipping, pattering, and kleptoparasitism. See Table S1 for both flight-style and foraging-style assignments.

### Skeletal Morphology and Analytical Approaches

**Figure 3:**
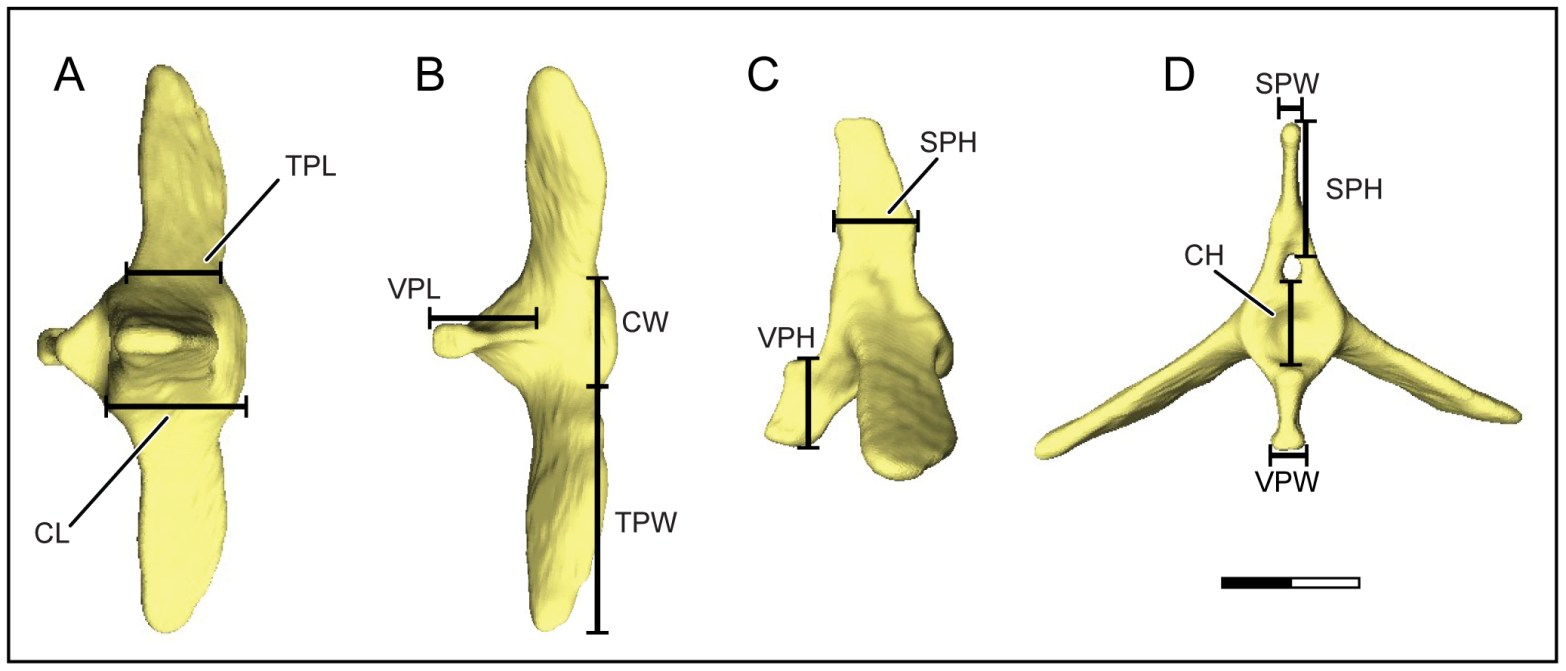
Free caudal vertebra in dorsal (A), ventral (B), left lateral (C), and anterior (D) views. Skeletal metrics collected: Centrum length (CL), centrum width (CW), centrum height (CH), transverse process length (TPL), transverse process width (TPW), spinous process length (SPL), spinous process width (SPW), spinous process height (SPH), ventral process length (VPL, ventral process width (VPW), ventral process height (VPH). Scale bar equals 2 mm.

In order to fully characterize caudal skeletal morphology, two datasets were collected. First, free caudal vertebral morphology was quantified using linear measurements. The following metrics were collected: centrum craniocaudal length, centrum width, centrum height, transverse process craniocaudal length, transverse process width, spinous process craniocaudal length, spinous process width, spinous process height, ventral process craniocaudal length, ventral process width, ventral process height (Fig. 3). These metrics were collected at three serial positions within the caudal vertebral series. The first (i.e. post-synacral) free caudal vertebra, the vertebra halfway along the length of the caudal series, and the last (i.e. propygostylar) free caudal vertebra. For individuals with an even number of free caudal vertebrae, the two middle vertebrae were measured and averaged. In order to take into account the effect of body size, the geometric mean of five additional measurements was used as a proxy for body size: sternal length, sternal width, height of sternal keel, synsacral length, and femur length [43], [67], [68]. Specimens and their institutional identification numbers are listed in Table S1. Linear measurements of free caudal vertebrae and body size proxies are provided in Table S2, averaged by species. A phylogenetic least-squares regression was conducted to correct raw measurements for body size, with the species’ means of the residuals used as variables for subsequent analyses [69], [70]. All linear measurements were obtained using digital calipers (Fowler digital calipers, Fred V. Fowler Company, Inc., Auburndale, MA).

The second dataset characterizes the morphology of the pygostyle using geometric morphometrics. Given that the pygostyle is irregularly shaped (Fig. 1, 2), laterally compressed, and lacks explicitly defined homologous landmarks, Elliptical Fourier Analysis (EFA) was used to quantify morphological variation in this structure. EFA is an outline analysis method commonly used on landmark-poor outline shapes [71]–[75].

**Figure 4:**
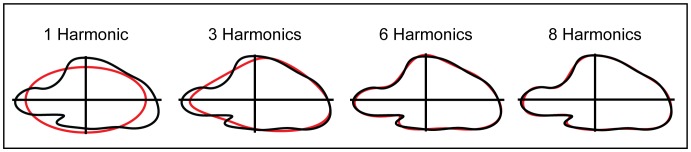
Outline reconstruction using Elliptical Fourier Descriptors. Black contours represent the original outline shape of the pygostyle of *Phoebastria immutabilis.* Red contours represented the reconstructed shape using the corresponding number of harmonics. Increasing the number of harmonics increases the detail of the reconstructed shape and the accuracy with which it approximates the true shape.

Fourier analysis utilizes a digitized outline of a shape consisting of a series of x and y coordinates for each pixel around the contour of a given shape. Separate Fourier decompositions are carried out for the change in the sequences of x- and y- coordinates around the perimeter. The result is a set of harmonically related (sine and cosine) equations, with each one referred to as a harmonic. For each harmonic, the sine and cosine equations describe the shape of an ellipse [71], [72]. Taken together, many harmonics may be used to describe more and more complex shapes (Fig. 4). The total number of variables (Fourier descriptors) is 4*n*, where *n* is the number of harmonics [71]. As with traditional, landmark based geometric morphometrics, the effects of size, position, and rotation must be removed such that only shape information remains. This is accomplished by standardizing Elliptical Fourier Descriptors by the first harmonic of each specimen. The resulting shape variables are referred to as Normalized Elliptical Fourier (NEF) descriptors [71], [72], [76]. This normalization process also reduces the number of variables to 4*n-*3. NEF coefficients can then be used as variables in multivariate statistical analyses.

In order to conduct the EFA, each specimen (Table S1) was photographed in lateral view (Fig. 2). Pygostyle outlines were digitized, Fourier transformed, and normalized using SHAPE v. 1.3 [77]. In order to remove superfluous variables from the dataset, the number of harmonics to retain was determined using the Fourier power method [71], [76]. For a given harmonic, *n*, Fourier power is calculated as
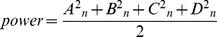



The number of harmonics retained is determined by the number required to obtain 99% of the cumulative power [71]. For the 160 pygostyle specimens photographed, eight harmonics comprise 99% of the cumulative power (Momocs R package, [78]). For each species, harmonic descriptors were averaged, resulting in 51 observations (species) and 37 variables (NEF descriptors).

### Phylogenetic Signal

Taxa in interspecific comparative studies cannot be treated as independent data points in statistical analyses because the phylogenetic relatedness of organisms introduces a degree of non-independence [79], [80]. The effect of phylogeny on caudal morphology was first quantified and then formally taken into account as part of each statistical approach.

For both the free caudal vertebrae and pygostyle datasets, phylogenetic signal was quantified using Pagel’s λ [81]. Pagel’s λ is a tree transformation parameter that measures the degree to which evolutionary relationships predict the observed patterns of variation/similarity in the data. This parameter varies between λ = 0 and λ = 1. If λ = 0, phylogenetic relatedness has no influence on the data and the tree can be transformed into a star phylogeny (equivalent to using ahistorical comparative methods). If λ = 1 the data fit a Brownian motion model of evolution given the original untransformed branch lengths. The optimal lambda for each dataset was calculated using the phytools R package [69], [82].

For the EFA dataset, an additional metric of phylogenetic signal was used. Calculating an optimal λ for a given dataset assumes that the data are multivariate. Shape data are in fact a single multidimensional character, and as such, is better served by calculating phylogenetic signal using the alternative ‘consistency index’ [83]. This metric varies from 0 to 1, where 0 = high homoplasy (low phylogenetic signal) and 1 = low homoplasy (high phylogenetic signal). The index is calculated using a permutation test. First, the amount of morphological change along the branches of the tree is calculated. Next, the shape data are shuffled among the tips of the tree and the amount of shape change is recalculated and compared to the observed value. If phylogeny has little effect, swapping the data among the tips will be equally likely to increase or decrease the amount of total tree length, and thus, on average not impart a noticeable effect. Conversely, if the effect of phylogeny is high, shuffling tip data are predicted to increase amount of change along the tree [83]. The consistency index for pygostyle shape was calculated using the geomorph package in R [84].

The higher-level phylogenetic relationships among the members of the “waterbird” and shorebird clades are somewhat contested [46]–[49], [85]. In order to take into account this phylogenetic uncertainty, each analysis was conducted using two alternative topologies, one using a “backbone” based on Hackett et al. [46] and the other using a “backbone” from Ericson et al. [49]. The former topology resolves Aequornithes as a monophyletic group, whereas the latter does not. The two phylogenetic hypotheses also differ in their placement of Phaethontidae. For each backbone topology, a sample of 5000 trees was obtained from the posterior distribution of trees on http://www.birdtree.org [48]. A Maximum Clade-Credibility (MCC) tree for each topology was produced using TreeAnnotator v1.6.2 [86]. The two MCC trees were used for all comparative analyses.

### Comparative Analyses

The two primary goals of the analyses conducted herein are to determine whether birds belonging to different ecological groups are characterized by different caudal skeletal morphology, and if so, identify which components of caudal skeletal morphology best explain differences among the groups. Phylogenetic MANOVAs (geiger R package; [87], [88]) were used to test for significant differences in morphology among functional groups. For the free caudal vertebrae dataset, separate tests were conducted for the first caudal vertebra, mid-caudal vertebra, and propygostylar vertebra. For the pygostyle shape dataset, the dimensionality of the data was first reduced by conducting a phylogenetic principal components analysis on an evolutionary variance-covariance matrix of the normalized Fourier descriptors [70]. Custom R scripts for computing and plotting phylogenetic PCA of elliptical Fourier data are provided in Supplementary File S1 The significant principal components (those that explain 5% or more of the total observed variance) were used as the dependent variables in the MANOVAs. MANOVAs were repeated using flight style and foraging style as the grouping factor and with both the Hackett et al. [46] backbone tree and Ericson et al. [49] backbone tree.

In order to determine which aspects of morphological variation best explain the differences among functional (flight or foraging) groups, we used a Phylogenetic Flexible Discriminant Analysis (pFDA), a multigroup classification tool related to Linear Discriminant Analysis and Canonical Correlation Analysis [89]–[91]. This method involves using a phylogenetic generalized least squares regression to construct a model estimating the relationship between the dependent variables (morphology) and group identity. The model is then used to predict group identity for each taxon given the data [89], [91]. The accuracy of the model–the degree to which group identify can be predicted by its morphology–can be evaluated by its misclassification rate. The misclassification rate equals the proportion of species that were improperly assigned to their respective class using the model (lower misclassification rate means higher accuracy of the model). Finally, the pFDA model can be used to generate an ordination plot to assist in the interpretation of the characters that differentiate each group. As with the MANOVAs, pFDA was repeated using both topologies and both eco-functional classification schemes.

## Results

### (a) Phylogenetic Signal Results

Phylogenetic relationships influence both free caudal vertebral anatomy and pygostyle shape. Pagel’s λ was slightly different for the first, middle, and last vertebra, but ranged between 0.418 and 0.723 (Table 1), thus the phylogenetic signal in free caudal vertebra can be characterized as moderate to high. Pagel’s λ was also calculated using the NEF descriptors for pygostyle shape and found to be 0.42, indicating a moderate degree of signal (Table 1). Using the consistency index, a more appropriate measure of phylogenetic signal for geometric morphometric data, phylogenetic signal for pygostyle shape was found to be approximately 0.45 (p<0.001), confirming a moderate level of phylogenetic influence on morphology. The results of the tests of phylogenetic signal were not substantially different when either of the two topologies were used, nor were the results of any of the subsequent analyses. As such, results are presented for the Hackett topology only [46]. These results justify the use of the phylogenetic comparative methods used bellow.

**Table 1:** Phylogenetic Signal.

Dataset	Pagel’s λ	Log Likelihood
First Vertebra	0.6786913	−432.4101
Middle Vertebra	0.53254101	−552.9049
Last Vertebra	0.7236591	−574.1383
Pygostyle Shape	0.4181343	5239.667

### (b) Phylogenetic MANOVA Results

The first, middle, and last free caudal vertebrae were analyzed using phylogenetic MANOVA for both topologies and for both eco-functional classification schemes (flight style and foraging style). In nearly all cases we found a significant difference in caudal vertebral anatomy among the groups (Table 2). Birds that utilize different flight styles differ in the dimensions of their first, middle, and last free caudal vertebrae (p<0.05), regardless of the choice of phylogenetic tree. Taxa that utilize different foraging styles have significantly different post-synsacral and pre-pygostylar vertebrae (p<0.05). Middle caudal vertebrae did not exhibit significant differences (p>0.1).

**Table 2:** Phylogenetic MANOVA Results: Free Caudal Vertebrae.

Dataset	Grouping	Degrees ofFreedom	Pillai-BartlettTrace	Approximate FNumber	Ahistoricalp-Value	Phylogeneticp-Value	Significance
First Vertebra	Flight Style	5	1.9958	3.4877	2.42E-09	0.002997	*
Middle Vertebra	Flight Style	5	2.136	2.6443	4.85E-07	0.02198	*
Last Vertebra	Flight Style	5	2.1851	2.7521	1.61E-07	0.01998	*
First Vertebra	Forging Style	4	1.632	3.6183	3.11E-08	0.03696	*
Middle Vertebra	Forging Style	4	1.8754	3.1297	1.04E-07	0.1179	
Last Vertebra	Forging Style	4	2.3282	4.9376	6.45E-14	0.000999	*

Phylogenetic MANOVAs were also used to examine whether different flight or foraging groups differ in pygostyle shape. The PC scores from a phylogenetic PCA were used as the dependent variables in the MANOVA. The PCA indicates that the first six PC axes combined explain >85% of the cumulative variance (>5% per axis), and these six axes were retained for the MANOVAs. Pygostyle shape does not differ significantly among flight style groups (p>0.1, Table 3). Among foraging groups, however, pygostyle shape is nearly significantly different (p = 0.05195). If the non-aquatic foraging groups (i.e., terrestrial and aerial) are combined, such that the groups are Plunge Dive, Foot-propelled Pursuit Dive, Wing-propelled Pursuit Dive, and Non-diving, the results of the phylogenetic MANOVA are significant at p<0.01 (Table 3).

**Table 3:** Phylogenetic MANOVA Results: Principal Components of Pygostyle Shape.

Grouping	Degrees ofFreedom	Pillai-BartlettTrace	Approximate FNumber	Ahistoricalp-Value	Phylogeneticp-Value	Significance
Flight Style	5	1.0598	1.9724	3.01E-03	0.3766	
Foraging Style	4	1.2827	3.4615	1.04E-06	0.05195	
Diving Type	3	1.1795	4.751	5.05E-08	0.008991	*

### (c) Phylogenetic FDA (pFDA) Results

**Figure 5:**
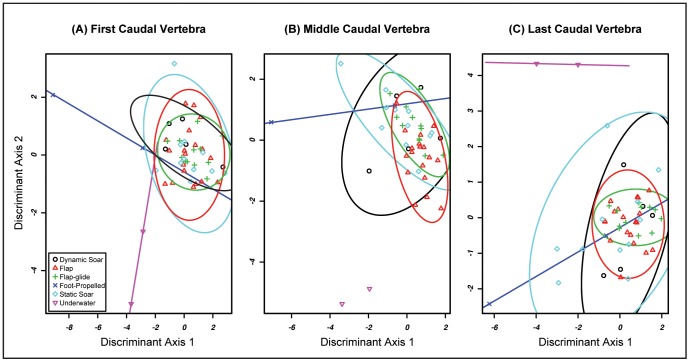
5a: Flight Style pFDA Plot: First Caudal Vertebra. Misclassification Rate = 41.18%. 5b: Flight Style pFDA Plot: Middle Free Caudal Vertebra. Misclassification Rate = 37.25%. 5c: Flight Style pFDA Plot: Last Free Caudal Vertebrae. Misclassification Rate = 37.25%.

To assist with interpreting which specific variables best explain differences among groups, we used pFDA ordinations. Using flight style as the grouping factor, pFDA of each of the three free caudal vertebrae generated a misclassification rate of 37–41% (Fig. 5). The majority of misclassifications occurred between flapping and flap-gliding taxa, in addition to commonly misclassifying both static and dynamic soaring taxa as flappers. In general, only wing-propelled flightless birds (*Pygoscelis papua* and *Pygoscelis adeliae*) and one foot-propelled flightless bird (*Phalacrocorax harrisi*) consistently occupy distinct regions of pFDA morphospace. *Pygoscelis* is characterized by a dorsoventrally restricted, laterally wide centrum and spinous process and a laterally restricted transverse process. *Phalacrocorax harrisi* exhibits a large spinous process and a small vertebral centrum. The remaining 48 taxa, representing the flap, flap-glide, static soar, and dynamic soar groups are clustered together in pFDA morphospace and lack any strong discriminating characteristics among the groups.

**Figure 6:**
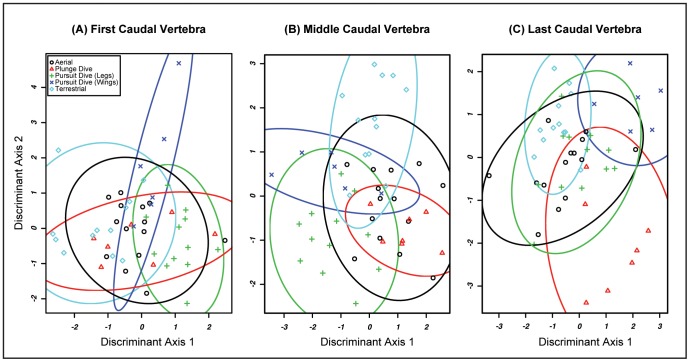
6a: Foraging Style pFDA Plot: First Free Caudal Vertebrae. Misclassification rate = 39.22%. 6b. Foraging Style pFDA Plot: Middle Free Caudal Vertebrae. Misclassification rate = 31.37%. 6c: Foraging Style pFDA Plot: First Last Caudal Vertebrae. Misclassification rate = 23.52%.

When foraging style is used as the grouping factor, the misclassification rate is 23–39% (Fig. 6). The highest misclassification rates for foraging style occur in the first and middle caudal vertebra datasets (39% and 31% respectively). In these datasets, aerial foragers and plunge-diving foragers were most commonly misclassified. Several plunge divers were misclassified as aerial or terrestrial foragers. Aerial foragers were most commonly misclassified as terrestrial, but were occasionally placed among the pursuit-diving or plunge-diving groups. The results (Figs. 6a–b) of these two pFDA analyses illustrate that terrestrial, foot-propelled diving, and wing propelled diving birds occupy somewhat distinct regions of morphospace, whereas aerial and plunge-diving birds occupy a common region of morphospace that overlaps with the other groups.

A misclassification rate of 24% for the propygostylar vertebra dataset is the least severe among the examined free caudal elements (Fig. 6c). The patterns observed here are somewhat different than for vertebrae positioned more cranially along the series. Aerial foragers are again the most frequently misclassified, sometimes being placed among the terrestrial or pursuit-diving foragers. There is considerably less classification error for the other foraging groups. When errors do occur, taxa are most often placed among the aerial foragers. Terrestrial, plunge-diving, and wing-propelled pursuit-diving foragers each group in distinct regions of the pFDA plots (Fig. 6c). Plunge-diving and wing-propelled pursuit-diving foragers are high on Discriminant Axis 1, indicating both groups have an craniocaudally restricted centrum and an craniocaudally restricted, yet wide ventral process. These groups are distinct from one another in that plunge divers score low on Discriminant Axis 2 (dorsoventrally expanded, narrow centrum, large spinous process, and small transverse process) but wing-propelled divers are high on Axis 2 (dorsoventrally restricted, wide centrum, small spinous process, and large transverse process). In contrast, terrestrial foragers are low on Axis 1 but high on Axis 2. This position in morphospace corresponds to a dorsoventrally restricted, elongate and wide centrum, large transverse process, and small spinous process. Aerial foragers and foot-propelled pursuit divers occupy an overlapping region of morphospace roughly centered on the origin.

**Figure 7:**
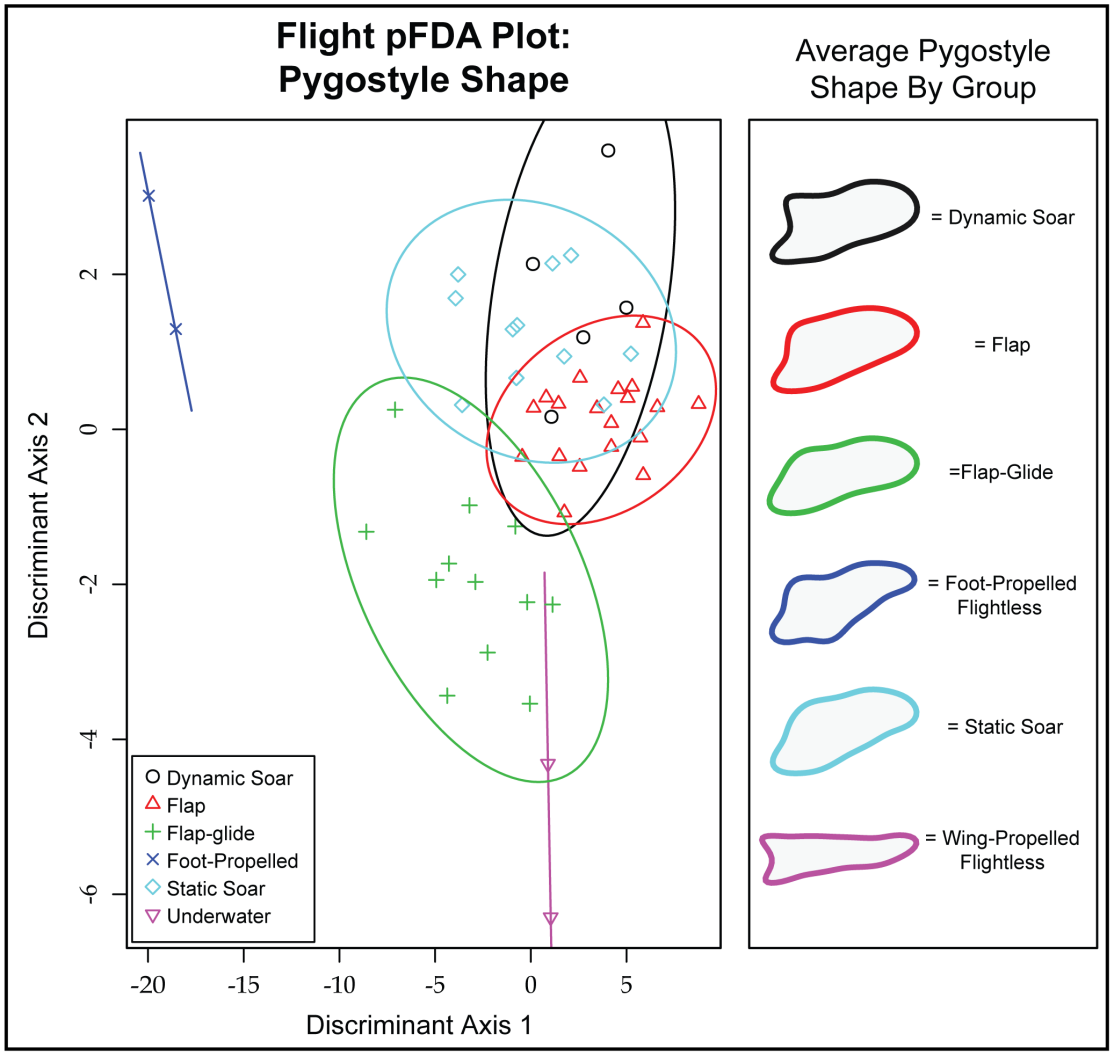
Flight Style pFDA Plot: Pygostyle Shape. Misclassification rate = 13.73%.

The misclassification rate for pFDAs of pygostyle shape data is much lower than for free caudal vertebrae. When flight style is used as the grouping factor, only seven out of 51 taxa are misclassified (13.7%). All seven of these cases involve ambiguous placements among flap, flap-glide, and static soaring taxa. As with free caudal vertebrae, wing-propelled flightless species (penguins) and foot-propelled flightless species (*Phalacrocorax harrisi* and *Rollandia microptera*) each occupy distinct regions of pFDA morphospace (Fig. 7). Foot-propelled flightless species score extremely low on Discriminant Axis 1 and moderately high on Axis 2, whereas wing-propelled flightless species score very low on Axis 2. Foot-propelled flightless birds have a pygostyle with a strong dorsal deflection and a pointed caudal margin. Wing-propelled flightless birds (penguins) have an extremely elongate, straight pygostyle. The remaining flight style groups (flap, flap-glide, static soar, dynamic soar) are somewhat restricted to a smaller region of morphospace, but overlap among these groups is minimal. Static soaring and flapping taxa have quite similar pygostyle shape, with a slight dorsal deflection and a slight taper. Static soarers have a slightly more pointed caudal margin whereas the typical flapping bird pygostyle is slightly rounded. Dynamic soaring birds have a rounded pygostyle with a deep articulation for the propygostylar vertebra. Flap-gliding taxa have a somewhat elongate, blunt pygostyle with a shallow articular surface.

**Figure 8:**
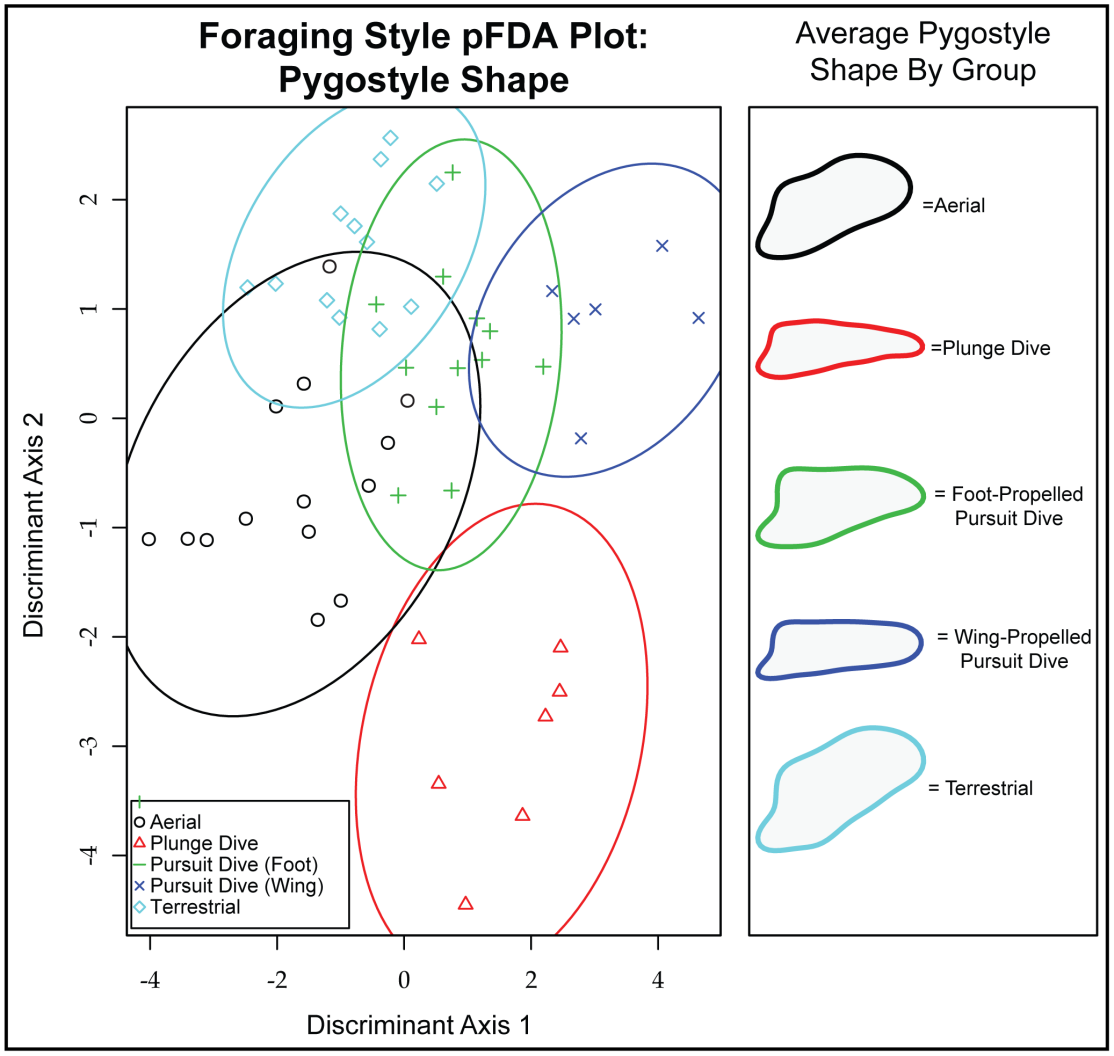
Foraging Style pFDA Plot: Pygostyle Shape. Misclassification rate = 3.92%.

Finally, the pFDA of pygostyle shape using foraging style as the grouping factor produces the most accurate discrimination, with just 2 of 51 taxa being misclassified (3.9%). The only misclassified taxa are the aerial foragers *Phoebastria immutabilis* and *Fregata magnificens*, both misclassified as terrestrial foragers. Their respective congeners, *Phoebastria nigripes* and *Fregata minor,* were correctly classified. A plot of the first two discriminant axes (Fig. 8) reveals that each foraging group is characterized by a distinct pygostyle shape. Plunge divers and wing-propelled pursuit divers both have a very elongate pygostyle; the plunge-diver pygostyle is tapered whereas wing-propelled pursuit divers are not. Foot-propelled pursuit divers are situated in pFDA phylomorphospace in between the wing-propelled divers and terrestrial foragers and exhibit a dorsoventrally expanded pygostyle that tapers to a point caudally. Terrestrial taxa have a pygostyle that is expanded dorsoventrally and is dorsocaudally directed. Finally, the average pygostyle of the aerial foraging group is similar to the terrestrial foraging condition, but exhibits a distinct narrowing midway along its length, giving an hourglass-like shape.

## Discussion

### Does Free Caudal Vertebral Morphology Differ Among Ecological Groups?

The two analyses, MANOVA and pFDA, give ostensibly conflicting results regarding the association between free caudal vertebral morphology and flight behavior (foraging and flight styles). Phylogenetic MANOVAs identified a significant difference in free caudal vertebral morphology among flight style groups. In contrast, the pFDAs of each free caudal vertebra using flight style as a grouping factor produced high misclassification rates. These conflicting results indicate that free caudal vertebral morphology is *not* useful for discriminating among flight style groups. Similar results were found using foraging style as a grouping factor – the MANOVAs for the first and last free caudal vertebrae return a significant difference among groups, yet pFDAs of these data result in a high misclassification rate.

The discordance between these results of the MANOVA and pFDA analyses is most likely attributed to the limitations of each analysis and the structure of the dataset. Whereas MANOVA is parametric, pFDA is not [88], [90], [91]. Thus, pFDA is more robust to deviations from multivariate normality and homoscedasticity than phylogenetic MANOVA. None of the free caudal vertebral datasets meet the criterion of multivariate normality. In order to mitigate the effects of the structure of the data, Pillai-Bartlett’s Trace was used as the test statistic because it is more robust to deviations from the assumptions of the MANOVA than the more common Wilk’s lambda test statistic [92]. The non-significant results of the pFDA suggest that the significant results of the phylogenetic MANOVA are spurious and that the structure of the free caudal vertebrae dataset is not well suited for such an analysis. The results of the pFDAs using these data can be interpreted with greater confidence.

Birds cannot be reliably assigned to flight style groups on the basis of free caudal vertebral morphology. The pFDA plots show that most taxa cluster together, with only the flightless birds *Pygoscelis spp.* and *Phalacrocorax harrisi* characterized by distinct caudal vertebral morphology compared to their close relatives. Although *Pygoscelis* and *Phalacrocorax harrisi* do not have similar free caudal morphology, the fact that these flightless swimming birds are distinct from all other sampled taxa suggests that this morphological divergence may be related to their specialized locomotor styles. The penguins possess a reduced transverse process and a wide, dorsoventrally compressed centum. In contrast, *P. harrisi* is characterized by a large spinous process with a small centrum, creating a dorsoventrally expanded, laterally compressed vertebra. The different vertebral anatomy in these clades of flightless taxa could be related to functional differences in tail use among wing-propelled flightless (*Pygoscelis*), foot-propelled (*Phalacrocorax*) flightless, and volant taxa. For example, the range of motion of the tail in elevation is limited by the “knocking together” of the spinous processes [18]. The tall spinous process of *P. harrisi* may thus restrict the extent to which it can elevate the tail. Given that tail elevation is observed primarily during takeoff [18], [86], it is possible that the unique caudal vertebral morphology of this taxon represents a relaxation of constraints maintaining the function of this structure as part of the flight apparatus.

Among the volant groups, most misclassification errors pertain to the flapping group. This manifests as either ambiguity between flapping and flap-gliding taxa or with dynamic- and static-soaring birds being classified in the flapping group. This suggests that flapping birds may have greater disparity in vertebral morphology than other flight style groups, and thereby occupy a greater region of pFDA morphospace. This is consistent with the hypothesis that birds with powerful wings for flight should display increased variance in tail form as constraints on the tail as a component of the aerial locomotor apparatus are relaxed [13].

When foraging style is used as the grouping factor for pFDA ordinations, the results are similar: high misclassification rates for first and middle caudal vertebrae and a moderate misclassification rate for the last caudal vertebrae. Misclassification error decreases and foraging groups separate better in phylomorphospace moving from cranial to caudal through the free vertebral series. The propygostylar vertebra has moderate predictive power with only 24% misclassification. The differences in predictive power among the three positions along the caudal vertebral column could be related to the association between more distal caudal vertebrae. The distal caudal vertebrae are variably ankylosed as part of the pygostyle and the distal-most free caudal vertebra articulates with the pygostyle. Baumel [18] noted that in rock dove (*Columba livia*) the propygostylar vertebra is reduced in size and hypothesized that this was an adaptation for increased freedom of movement at the propygostylar joint. Given that pygostyle shape seems to be influenced by flight behavior (see below) and that the propygostylar vertebra is functionally linked with pygostyle, it is possible that the same evolutionary forces drive pygostyle and propygostylar vertebral morphological variation but do not influence more cranial regions of the caudal series.

### Does Pygostyle Shape Differ Among Ecological Groups?

In contrast to free caudal vertebral morphology, pygostyle shape is an excellent predictor of foraging style in waterbirds. The results of the phylogenetic MANOVAs and pFDAs are more congruent with one another using NEF descriptors of pygostyle shape. Each foraging group is characterized by a significantly different pygostyle shape (Fig. 8). Aerial foragers exhibit a vertically-deflected pygostyle with a blunt caudal margin and dorsoventral constriction midway along its length, resulting in a distinctive hourglass shape. Terrestrial foragers have a generally similar pygostyle shape when compared to aerial foragers, but lack the dorsoventral constriction. Foot-propelled pursuit divers have a pygostyle that is dorsoventrally expanded at the cranial end but that tapers to a point caudally. Wing-propelled pursuit divers exhibit an exceptionally long pygostyle that does not taper. Plunge divers have a generally similar pygostyle, but one that tapers gradually. In general, underwater foragers (plunge dive and pursuit dive) have straight, elongate pygostyles, whereas birds that do not forage underwater (aerial and terrestrial) have craniocaudally restricted, dorsally-oriented pygostyles. The only misclassifications occurred between non-aquatic groups, supporting a dichotomy between aquatic and non-aquatic foragers.

**Figure 9:**
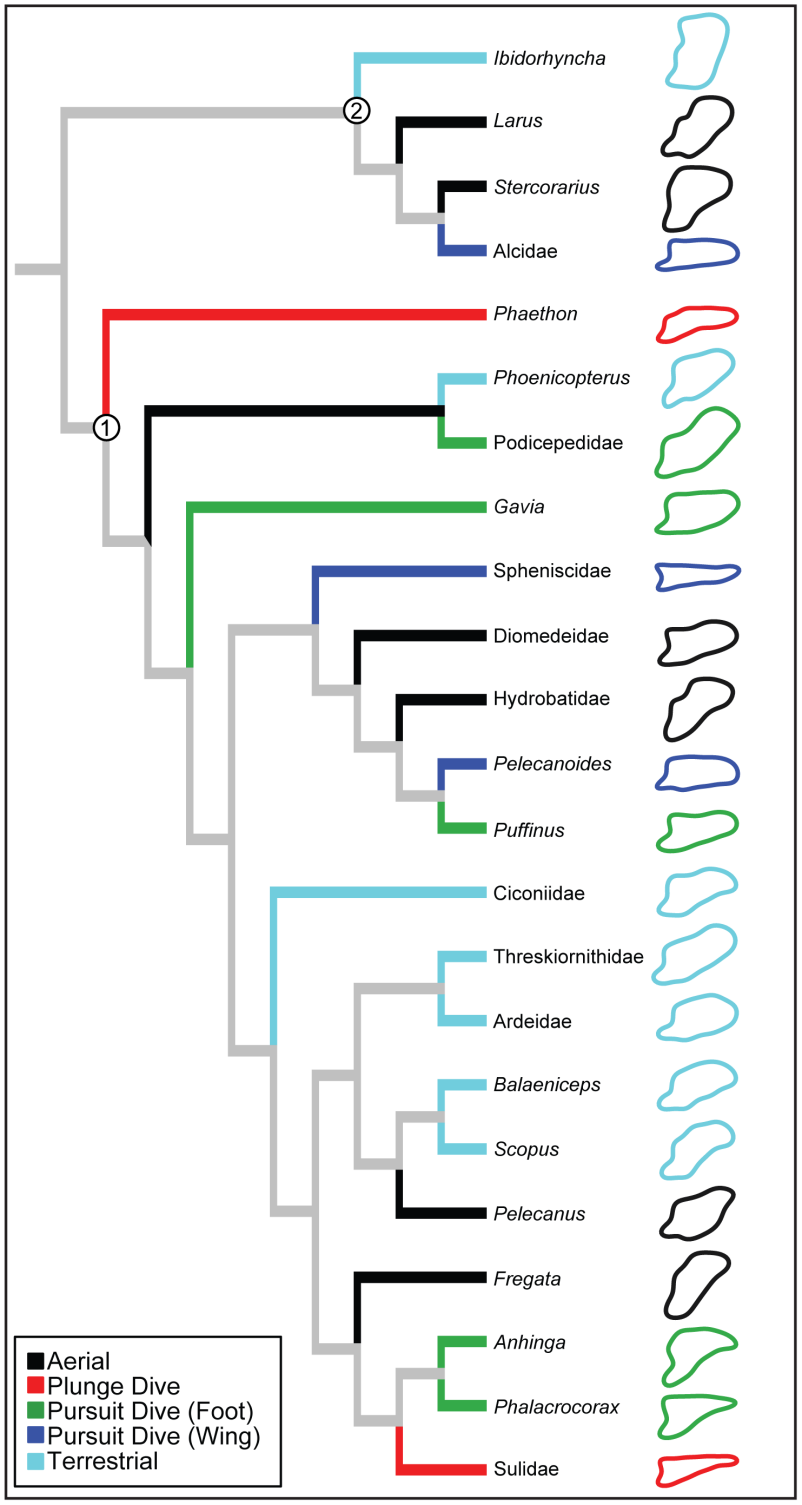
Foraging style and pygostyle shape mapped onto the phylogenetic topology of Hackett [46], [48]. Branch colors represent foraging style; internal branches are colored gray to indicate that ancestral foraging style is uncertain. Node 1, Aequornithes (waterbirds); Node 2, Charadriiformes (shorebirds).

Underwater foraging birds exhibit convergence in pygostyle morphology (Fig. 9). The significance of a straight, elongate pygostyle is likely related to the mechanical demands on the tail when moving through water as opposed to air. Observational data on captive Great Cormorants (*Phalacrocorax carbo*) and several species of penguins indicate that the tail is used as a steering structure, controlling pitch and yaw for high speed underwater turns [93]–[95]. Stifftail ducks (Oxyurinae), a group of foot-propelled diving specialists, also use the tail as a rudder [37], [96]. Quantitative data on the use of the tail in underwater locomotion in other birds is not available. Nonetheless, the ubiquitous use of the tail as a control surface in aerial locomotion and the observed use of the tail during swimming in certain clades suggest that the tail is no doubt an important part of the swimming locomotor apparatus in diving birds. An elongate pygostyle may confer some advantage when moving the tail through water.

Underwater locomotion imposes certain unique challenges. During flight, the wings and tail produce lift, resisting the downward force of gravity. Conversely, while diving underwater a bird must counteract its own buoyancy, an upward force pulling it toward the surface. Accordingly, wing-propelling diving birds use different power strokes for flying and swimming- the flight stroke produces a downward force whereas the swim stroke produces an upward force [97]–[100]. Birds that are capable of flight and underwater diving thus experience both dorsally and ventrally directed forces acting on the tail, whereas birds that do not dive experience primarily dorsally directed force (lift). This may influence the difference in pygostyle morphology between underwater foragers and aerial/terrestrial foragers: underwater foragers exhibit a dorsoventrally symmetrical pygostyle whereas aerial and terrestrial taxa possess an asymmetrical, dorsally deflected pygostyle (Fig. 8). Perhaps birds that utilize underwater locomotion require more symmetrical attachments for dorsiflexor and ventroflexor musculature, resulting in a more symmetrical pygostyle. Alternatively, an elongate, straight pygostyle may be related to resisting biomechanical forces rather than supplying muscle attachments. Diving birds such as alcids and penguins exhibit specialized limb bone geometry being better suited to resisting the high bending and torsional forces associated with the denser medium of water [101]. The geometry of the pygostyle of aquatic birds may similarly be able to resist such forces. These hypotheses require comparative surveys of caudal muscle anatomy (e.g., cross sectional area, pennation, fiber type) and pygostyle mechanical properties.

Another possible consequence of the evolution of a long, straight pygostyle is the orientation of the rectrices. Baumel [18] noted variability in the degree of concavity of the tail fan. Pigeons and some other taxa have medial rectrices that are positioned dorsally within the rectricial bulb relative to lateral rectrices, such that the array of tail feathers forms a “vaulted” or “tented” arrangement [18]. Other taxa, such as *Anser*, *Ardea*, *Chaetura*, and *Quiscalis* have a flat arrangement of the rectrices, with the rachises of each tail feather lying roughly on the same plane [18]. A dorsally-oriented pygostyle may facilitate the dorsoventral stacking of rectrices in birds with a tented tail whereas a straight pygostyle may be indicative of flat tail fan. Conformation of a link between pygostyle shape and rectricial configuration will require an extensive survey of soft tissue morphology. Additionally, the functional consequences of a tented tail are not currently known, as aerodynamic models of the avian tail assume a flat tail fan [25].

Convergent caudal morphology in diving birds is not surprising given the numerous morphological specializations observed in these forms. Diving waterbirds and anseriforms have a reduced level of skeletal pneumaticity relative to their non-diving relatives [50], [102]. Foot-propelled diving birds have pelvic girdle and hind limb morphology that increases mechanical advantage for paddling [96]. Diving pelecaniform birds (anhingas, cormorants), have distinct forelimb cross-sectional geometry with high levels of cortical bone, likely related to buoyancy modulation [42]. Similarly, the humerus of wing-propelled divers such as penguins and alcids exhibits thick cortical bone, making this element resistant to bending and torsion under the high mechanical loads involved with flapping underwater [101]. Foot-propelled divers typically exhibit a suite of traits related to increasing swimming performance such as a long, narrow pelvis, a large, stable knee articulation, and a posteriorly placed hip joint [44]. Taken together with the results of this study, it is clear that the evolution of diving behavior in birds results in a wide range of morphological adaptations to cope with the unique demands of underwater locomotion.

Finally, the high predictive power of the pFDA of pygostyle shape suggests that pygostyle morphology may be useful for interpreting the ecology of extinct pygostylian birds. Past studies have used forelimb, hind limb, and furcula morphology to predict ecology (flight style and/or foraging style) in extinct birds with some success [14], [44], [103], [104]. Incorporating information from the tail with data from the other two avian locomotor modules (wings and legs) could improve inferences of foraging behavior from the fossil record. For example, the Cretaceous diving bird *Baptornis* is characterized by an elongate pygostyle [105].

## Conclusions

Pygostyle shape is an excellent predictor of foraging style in waterbirds. Underwater foraging birds, such as cormorants, penguins, puffins, gannets, and tropicbirds, exhibit convergent evolution toward a strait, elongate pygostyle (Fig. 9). Moreover, each underwater foraging group (foot propelled, wing propelled, and plunge diving) has a distinctive pygostyle shape (Fig. 8). Free caudal vertebral morphology, in contrast, is a less informative predictor of flight style or foraging style groups. These results contribute to the body of knowledge on how the acquisition of underwater locomotor behaviors influences avian morphology. The tail skeleton, much like the forelimbs, hind limbs, and skeletal pneumaticity, is modified in swimming birds.

The disassociation of the tail module from the hind limb module in basal birds is thought to have been an important innovation that allowed for ecological diversification [17], [106], [107]. Each of the three locomotor modules (wings, legs, and tail) can evolve semi-independently and have been emphasized to varying degrees, increasing the diversity of locomotor repertoires available to birds [16], [17], [106], [107]. The use of the tail for locomotion is predicted to be emphasized in birds that are capable of complex flight behavior with small bodies and elevated nests [106]. The importance of the tail module in diving waterbirds with colonial nesting and large bodies is previously unrecognized. The diversification of diving birds may have been facilitated by evolution of caudal structure and function for underwater locomotion in the same way that diversification into other niches is thought to be related to correlated evolution of the wings and tail for aerial locomotion.

## Supporting Information

Table S1 **Taxon list.** Flight-style and foraging-style assignments, sample size, and specimen lists. Institutional Abbreviations– AMNH, American Museum of Natural History, New York, New York; CMNH, Carnegie Museum of Natural History, Pittsburgh, PA; FMNH, Field Museum of Natural History, Chicago, IL; NMNH, National Museum of Natural History, Washington, DC; OUVC, Ohio University Vertebrate Collection, Athens, OH.
(XLSX)

Table S2 **Skeletal Data Table.** Averaged by species.
(XLSX)

File S1 **R Script for Conducting Phylogenetic PCA Using EFA Data.**
(DOC)
