# Supplementary material for: Ecology and Caudal Skeletal Morphology in Birds: The Convergent Evolution of Pygostyle Shape in Underwater Foraging Taxa
Source: PLoS One. 2014 Feb 26;9(2):e89737. doi: 10.1371/journal.pone.0089737 (PMC3935938; doi:10.1371/journal.pone.0089737)
Supplement: File S1 — R Script for Conducting Phylogenetic PCA Using EFA Data. (DOC) [file pone.0089737.s003.doc]

Supplemental Data: R Script for Conducting Phylogenetic PCA Using EFA Data

**R Script:**

#####R Script: efa.phyl.pca.R

require(Momocs)

require(ape)

require(geiger)

require(phytools)

require(calibrate)

####

#the first two functions, NEF2COE and pca2shp.new, are slightly #modified versions of functions from the Momocs R package [1] for compatibility with the SHAPE software package [2] and the rest of the efa.phyl.pca.R functions.

####

NEF2COE takean input NEF file of normalized elliptical Fourier descriptors (as created with SHAPE) and outputs a COE object for use with the subsequent functions.

####

NEF2COE <-

function (nef.path)

{

nef <- readLines(nef.path)

HARMO.l <- grep(pattern = "HARMO", nef)

nb.h <- as.numeric(substring(nef[HARMO.l], 8))

nef <- nef[-(1:HARMO.l)]

nb.coo <- length(nef)/(nb.h + 1)

coo.i <- 1:nb.coo

coo.beg <- (coo.i - 1) * (nb.h + 1) + 1

coo.end <- coo.beg + nb.h

res <- matrix(NA, nrow = nb.coo, ncol = nb.h * 4, dimnames = list(nef[coo.beg], paste(rep(LETTERS[1:4], each = nb.h), 1:nb.h, sep = "")))

for (i in seq(along = coo.i)) {

nef.i <- nef[(coo.beg[i]+1):coo.end[i]]

x <- as.numeric(unlist(strsplit(nef.i, " ")))

x1<-x[!is.na(x)]

a.i<-x1[seq(1,length(x1),4)]

b.i<-x1[seq(2,length(x1),4)]

c.i<-x1[seq(3,length(x1),4)]

d.i<-x1[seq(4,length(x1),4)]

res[i, ]<-c(a.i,b.i,c.i,d.i)

}

return(Coe(res,method="eFourier"))

}

####

#pca2shp.new is an internal function used elsewhere and is not intended for use by the end user

####

pca2shp.new <-

function (pos, rot, mean.shp, method = c("efourier", "rfourier",

"tfourier"), scale = 1, amp = 1, trans = TRUE, nb.pts = 300,

rotate.shp)

{

if (!is.matrix(pos))

pos <- as.matrix(pos)

if (ncol(pos) != ncol(rot))

stop("rot an pos must have the same ncol")

if (length(mean.shp) != nrow(rot))

stop("mean.shp length must equals the col number of rot")

if (missing(method)) {

warning("Method not provided. efourier is used.")

p <- 1

method.i <- efourier.i

}

else {

p <- pmatch(tolower(method), c("efourier", "rfourier",

"tfourier"))

if (is.na(p)) {

warning("Unvalid method. efourier is used.")

}

else {

method.i <- switch(p, efourier.i, rfourier.i, tfourier.i)

}

}

mprod <- function(m, s) {

res <- m

for (i in 1:ncol(m)) {

res[, i] <- m[, i] * s[i]

}

return(res)

}

nb.h <- length(mean.shp)/ifelse(p == 1, 4, 2)

n <- nrow(pos)

res <- array(NA, dim = c(nb.pts, 2, n), dimnames = list(paste0("pt",

1:nb.pts), c("x", "y"), paste0("shp", 1:n)))

for (i in 1:n) {

ax.contrib <- mprod(rot, pos[i, ]) * amp

coe <- mean.shp + apply(ax.contrib, 1, sum)

if (p == 1) {

xf <- list(an = coe[1:nb.h + 0 * nb.h], bn = coe[1:nb.h +

1 * nb.h], cn = coe[1:nb.h + 2 * nb.h], dn = coe[1:nb.h +

3 * nb.h])

}

else {

xf <- list(an = coe[1:nb.h + 0 * nb.h], bn = coe[1:nb.h +

1 * nb.h])

}

coo <- l2m(method.i(xf, nb.h = nb.h, nb.pts = nb.pts))

coo <- coo.template(coo, size = scale)

if (!missing(rotate.shp)) {

coo <- coo.rotate(coo, rotate.shp)

}

if (trans) {

coo <- coo.trans(coo, pos[i, 1], pos[i, 2])

}

res[, , i] <- coo.force2close(coo)

}

invisible(res)

}

####

#neftrimmer is a function that removes the constants A1, B1, and C1 #from the matrix of normalized fourier coefficients before statistical #analysis

####

neftrimmer <-

function(nef){

c<-ncol(nef)

nef[,-c(1,(c/4+1),(c/2+1))]

}

####

#function to calculate phylogenetic PCA of EFA data [3]

####

efa.phyl.pca <-

function (tree, nefmat, nharm, method = "BM", mode = "cov")

{

if (class(tree) != "phylo")

stop("tree must be an object of class 'phylo.'")

if (colnames(nefmat[,c(1,ncol(nefmat)/4+1,ncol(nefmat)/2+1)] != c("A1","B1","C1")))

stop("nefmat must be a complete set of NEF descriptors")

if (length(strsplit(mode, split = "")[[1]]) <= 2) {

message(paste("mode = '", mode, "' not a valid option; setting mode = 'cov'", sep = ""))

mode = "cov"

}

if (all(strsplit(mode, split = "")[[1]] == strsplit("correlation",

split = "")[[1]][1:length(strsplit(mode, split = "")[[1]])]))

mode = "corr"

else if (all(strsplit(mode, split = "")[[1]] == strsplit("covariance",

split = "")[[1]][1:length(strsplit(mode, split = "")[[1]])]))

mode = "cov"

else {

message(paste("mode = '", mode, "' not a valid option; setting mode = 'cov'",

sep = ""))

mode = "cov"

}

nefmat<-nefmat[,c(1:nharm,(ncol(nefmat)/4+1):(ncol(nefmat)/4+nharm),(ncol(nefmat)/2+1):(ncol(nefmat)/2+nharm),((3*ncol(nefmat)/4)+1):(((3*ncol(nefmat))/4)+nharm))]

p<-ncol(nefmat)

Y<-neftrimmer(nefmat)

n <- nrow(Y)

m <- ncol(Y)

if (n > length(tree$tip))

stop("number of rows in NEF matrix cannot be greater than number of taxa in your tree")

Y <- as.matrix(Y)

if (is.null(rownames(Y))) {

if (nrow(Y) == n) {

print("NEF matrix has no names. function will assume that the row order of NEF matrix matches tree$tip.label")

rownames(Y) <- tree$tip.label

}

else stop("NEF matrix has no names and does not have the same number of rows as tips in tree")

}

else if (length(setdiff(rownames(Y), tree$tip.label)) !=

0)

stop("NEF matrix has rownames, but some rownames of Y not found in tree")

C <- vcv.phylo(tree)[rownames(Y), rownames(Y)]

if (method == "BM") {

temp <- phyl.vcv(Y, C, 1)

V <- temp$R

a <- t(temp$alpha)

C <- temp$C

}

else if (method == "lambda") {

temp <- optimize(f = likMlambda, interval = c(0, maxLambda(tree)),

X = Y, C = C, maximum = TRUE)

lambda <- temp$maximum

logL <- as.numeric(temp$objective)

temp <- phyl.vcv(Y, C, lambda)

V <- temp$R

a <- t(temp$alpha)

C <- temp$C

}

invC <- solve(C)

if (mode == "corr") {

Y = Y/matrix(rep(sqrt(diag(V)), n), n, m, byrow = T)

V = V/(sqrt(diag(V)) %*% t(sqrt(diag(V))))

a <- matrix(colSums(invC %*% Y)/sum(invC), m, 1)

}

es = eigen(V)

result <- list()

result$mean.shp<-colMeans(nefmat)

result$num.harms<-nharm

result$Eval <- diag(es$values)

result$Evec <- es$vectors

dimnames(result$Eval) <- list(paste("PC", 1:ncol(Y), sep = ""),

paste("PC", 1:ncol(Y), sep = ""))

dimnames(result$Evec) <- list(colnames(Y), paste("PC", 1:ncol(Y),

sep = ""))

A <- matrix(rep(a, n), n, m, byrow = T)

result$S <- (Y - A) %*% result$Evec

Ccv <- t(Y - A) %*% invC %*% result$S/(n - 1)

result$L <- matrix(, m, m, dimnames = list(colnames(Y), paste("PC",

1:ncol(Y), sep = "")))

for (i in 1:m) for (j in 1:m) result$L[i, j] <- Ccv[i, j]/sqrt(V[i,

i] * result$Eval[j, j])

if (method == "lambda") {

result$lambda <- lambda

result$logL.lambda <- logL

}

A1<-rep(0,m)

B1<-rep(0,m)

C1<-rep(0,m)

result$L.full<-rbind(A1,result$L[1:((p/4)-1),],B1,result$L[(p/4):((p/2)-2),],C1,result$L[((p/2)-1):(p-3),])

result$Evec.full<-rbind(A1,result$Evec[1:((p/4)-1),],B1,result$Evec[(p/4):((p/2)-2),],C1,result$Evec[((p/2)-1):(p-3),])

result$var.contrib<-(diag(result$Eval)/sum(result$Eval))*100

result$sig.PCs <- sum((result$var.contrib>5))

result$phy<-tree

return(result)

}

###

#function to estimate phylogenetic signal using the consistency index. #Code is modified from geomorph to utilize EFA data [4].

###

efa.phylo.signal <-

function(phy,A,nharm,iter=999)

{

require(ape)

require(geiger)

N <- length(phy$tip.label)

A <- A[phy$tip.label, ]

A <- A[, c(1:nharm, (ncol(A)/4 + 1):(ncol(A)/4 +

nharm), (ncol(A)/2 + 1):(ncol(A)/2 + nharm),

((3 * ncol(A)/4) + 1):(((3 * ncol(A))/4) +

nharm))]

A<- neftrimmer(A)

SSC.o <- NULL

anc.states <- matrix(NA, nrow = (nrow(A) - 1), ncol = ncol(A))

for (i in 1:ncol(A)) {

anc.states[, i] <- fastAnc(phy, A[, i])

}

dist.mat <- as.matrix(dist(rbind(as.matrix(A), as.matrix(anc.states)))^2)

SSC.o<-0

for (i in 1:nrow(phy$edge)) {

SSC.o <- SSC.o + dist.mat[phy$edge[i, 1], phy$edge[i, 2]]

}

P.val <- 1

for (i in 1:iter) {

A.r <- A[sample(nrow(A)), ]

row.names(A.r) <- row.names(A)

SSC.r <- NULL

anc.states <- matrix(NA, nrow = (nrow(A) - 1), ncol = ncol(A))

for (i in 1:ncol(A.r)) {

anc.states[, i] <- fastAnc(phy,A.r[, i])

}

dist.mat.r <- as.matrix(dist(rbind(as.matrix(A.r), as.matrix(anc.states)))^2)

SSC.r <- 0

for (i in 1:nrow(phy$edge)) {

SSC.r <- SSC.r + dist.mat.r[phy$edge[i, 1], phy$edge[i,2]]

}

P.val <- ifelse(SSC.r <= SSC.o, P.val + 1, P.val)

}

P.val <- P.val/(iter + 1)

return(list(phy.signal = SSC.o, pvalue = P.val))

}

####

# phyl.efa.morphospace is a simple function for plotting the results of the phylogenetic PCA of EFA data. It is a modified version of the morpho.space function of the Momocs packageb [1].

####

phyl.efa.morphospace <-

function (pca, xax = 1, yax = 2, xlim, ylim, nb.pts = 300, pos.shp = c("li",

"circle", "range")[3], nr.shp = 6, nc.shp = 5, amp.shp = 1,

scale.shp = 1, rotate.shp = 0, circle.nb.shp = 12, circle.r.shp,

plot = TRUE, layer = TRUE, col.shp = "#70809011", border.shp = "#708090",

pch.pts = 20, col.pts = "grey40", first.point = FALSE)

{

if (is.data.frame(pos.shp))

pos.shp <- as.matrix(pos.shp)

if (is.matrix(pos.shp)) {

if (ncol(pos.shp) != 2) {

stop("When passed with a matrix, pos.shp requires a two columns matrix")

}

pos <- pos.shp

}

else if (pos.shp == "li") {

pos <- pca$S[, c(xax, yax)]

}

else if (pos.shp == "circle") {

if (missing(circle.r.shp)) {

li.2 <- apply(pca$S[, c(xax, yax)], 2, function(x) x^2)

li.len <- apply(li.2, 1, function(x) sqrt(sum(x)))

circle.r.shp <- mean(li.len)

}

t <- seq(0, 2 * pi, len = circle.nb.shp + 1)[-(circle.nb.shp +

1)]

pos <- cbind(circle.r.shp * cos(t), circle.r.shp * sin(t))

}

else if (pos.shp == "range") {

pos <- expand.grid(seq(min(pca$S[, xax]), max(pca$S[,

xax]), len = nr.shp), seq(min(pca$S[, yax]), max(pca$S[,

yax]), len = nc.shp))

pos <- as.matrix(pos)

}

else {

stop("shp.pos must be passed with values li, circle, range or a matrix of coordinates")

}

if (missing(scale.shp)) {

scale.shp <- min(apply(pca$S[, c(xax, yax)], 2, function(x) diff(range(x)))/(c(nr.shp,

nc.shp) - 1))

}

shapes <- pca2shp.new(pos, rot = pca$Evec.full[, c(xax, yax)], mean.shp = pca$mean.shp,

method = "efourier", scale = scale.shp, amp = amp.shp,

rotate.shp = rotate.shp, nb.pts = nb.pts)

if (plot) {

if (missing(xlim) & missing(ylim)) {

w <- apply(shapes, 2, range)

}

else {

w <- cbind(xlim, ylim)

}

op <- par(no.readonly = TRUE)

on.exit(par(op))

par(mar = c(3, 3, 1, 1))

plot(pca$S[, c(xax, yax)], xlim = w[, 1], ylim = w[,

2], asp = 1, las = 1, col = col.pts, pch = pch.pts,

cex = 1, cex.axis = 0.7, ann = FALSE)

abline(h = 0, v = 0, lty = 2, col = "grey80")

box()

}

if (layer) {

apply(shapes, 3, coo.draw, points = FALSE, border = border.shp,

col = col.shp, first.point = first.point)

}

invisible(shapes)

}

####

# phylo.efa.plot is a very powerful function for plotting the results from efa.phyl.pca. It combines the dudi.plot function of Momocs [1] and the phylomorphospace function of phytools [5].

####

phylo.efa.plot <-

function (pca, fac = NULL, groupings= NULL, xax = 1, yax = 2, grid = TRUE, points = TRUE,

pch.points = 1, col.points = "black", cex.points = 0.8, labels = FALSE,

label = rownames(pca$S), boxes = TRUE, clabel = 0.6, neighbors = FALSE,

draw.tree = FALSE, col.nei = "grey90", lwd.nei = 0.5, star = FALSE,

col.star = "grey60", cstar = 1, ellipses = FALSE, col.ellipse = "grey30",

cellipse = 1, axesell = TRUE, chull = FALSE, col.chull = "grey30",

optchull = c(0.5, 1), arrows = FALSE, edge.arrow = FALSE,

box.arrow = TRUE, maxnb.arrow = 10, dratio.arrow = 0.2, shapes = TRUE,

pos.shp = c("li", "circle", "range", "full")[3], nr.shp = 6,

nc.shp = 5, amp.shp = 1, scale.shp = 0.666, first.point.shp = FALSE,

rotate.shp = 0, circle.nb.shp = 12, circle.r.shp, col.shp = "#70809011",

border.shp = "#708090", rug = TRUE, rug.ticksize = 0.01,

rug.col = "#708090", eigen = FALSE, eigen.ratio = 0.2, palette = col.sari,

title = substitute(pca), center.orig = FALSE, zoom.plot = 1,

control = list(), tree.lab = FALSE)

{

if (!missing(fac)) {

if (!is.factor(fac)) {

if (ncol(groupings) == 0) {

fac <- factor(rep("", nrow(pca$S)))

}

else {

groupings<-groupings[row.names(pca$S),]

fac <- groupings[, fac]

}

}

}

if ((nlevels(fac) > 1)) {

if (missing(col.star))

col.star <- paste(palette(nlevels(fac)), "33", sep = "")

if (missing(col.ellipse))

col.ellipse <- palette(nlevels(fac))

if (missing(col.chull))

col.chull <- palette(nlevels(fac))

}

if (center.orig) {

li.2 <- apply(pca$S[, c(xax, yax)], 2, function(x) x^2)

li.len <- apply(li.2, 1, function(x) sqrt(sum(x)))

w <- max(li.len) * (1/zoom.plot)

s.label(pca$S, xax = xax, yax = yax, xlim = c(-w, w),

clabel = 0, cpoint = 0, sub = title, grid = grid)

}

else {

s.label(pca$S, xax = xax, yax = yax, clabel = 0, cpoint = 0,

sub = title, grid = grid)

}

xaxp <- par("xaxp")

ax <- (xaxp[2] - xaxp[1])/xaxp[3]

yaxp <- par("yaxp")

ay <- (yaxp[2] - yaxp[1])/yaxp[3]

d <- min(ax, ay)

op <- par("mar")

par(mar = rep(0.1, 4))

if (rug) {

rug(pca$S[, xax], side = 1, ticksize = rug.ticksize,

col = rug.col, lwd = 0.4)

rug(pca$S[, yax], side = 2, ticksize = rug.ticksize,

col = rug.col, lwd = 0.4)

box()

}

if (neighbors) {

fun <- function(x, coo, col, lwd) {

segments(coo$x[x[1]], coo$y[x[1]], coo$x[x[2]], coo$y[x[2]],

col = col, lwd = lwd)

}

neig <- nb2neig(tri2nb(pca$S[, c(xax, yax)]))

coo <- list(x = pca$S[, xax], y = pca$S[, yax])

apply(unclass(neig), 1, fun, coo = coo, col = col.nei,

lwd = lwd.nei)

}

if (star & !is.null(fac)) {

s.class(pca$S, xax = xax, yax = yax, fac = fac, clabel = 0,

cpoint = 0, add.plot = TRUE, cstar = cstar, col = col.star,

cellipse = 0)

}

if (ellipses & !is.null(fac)) {

s.class(pca$S, xax = xax, yax = yax, fac = fac, clabel = 0,

cpoint = 0, add.plot = TRUE, cstar = 0, col = col.ellipse,

cellipse = cellipse, axesell = axesell)

}

if (chull & !is.null(fac)) {

s.chull(pca$S, xax = xax, yax = yax, fac = fac, col = col.chull,

optchull = optchull, add.plot = TRUE)

}

if (arrows) {

arr.2 <- apply(pca$L.full[, c(xax, yax)], 2, function(x) x^2)

arr.len <- apply(arr.2, 1, function(x) sqrt(sum(x)))

if (maxnb.arrow > ncol(pca$mean.shp)) {

maxnb.arrow <- ncol(pca$mean.shp)

}

arr.sorted <- order(arr.len, decreasing = TRUE)[1:maxnb.arrow]

arr.disp <- if (missing(dratio.arrow)) {

arr.len[arr.sorted] > 0

}

else {

arr.len[arr.sorted] > d * dratio.arrow

}

if (sum(arr.disp) > 0) {

arr.co <- pca$L.full[names(which(arr.disp)), c(xax,

yax)]

s.arrow(arr.co, 1, 2, label = rownames(arr.co), edge = edge.arrow,

add.plot = TRUE, boxes = box.arrow, clabel = clabel)

}

}

if (shapes) {

if (!is.matrix(pos.shp)) {

if (pos.shp == "full") {

w <- par("usr")

pos.shp <- as.matrix(expand.grid(seq(w[1] + d/2,

w[2] - d/2, len = nr.shp), seq(w[3] + d/2,

w[4] - d/2, len = nc.shp)))

}

}

shapes <- phyl.efa.morphospace(pca, xax = xax, yax = yax,

plot = FALSE, layer = TRUE, pos.shp = pos.shp, nr.shp = nr.shp,

nc.shp = nc.shp, amp.shp = 1, scale.shp = d * scale.shp,

rotate.shp = rotate.shp, circle.nb.shp = circle.nb.shp,

circle.r.shp = circle.r.shp, col.shp = "#70809011",

border.shp = "#708090", first.point = first.point.shp,

pch.pts = NA)

}

if (points) {

repeach <- function(x, each) {

if (length(x) != length(each))

return(rep(x[1], sum(each)))

res <- vector(mode = class(x[1]))

for (i in seq(along = x)) {

res <- append(res, rep(x[i], each[i]))

}

return(res)

}

if (!is.null(fac)) {

nb <- table(fac)

pch.points <- repeach(pch.points, nb)

if (missing(col.points)) {

col.points <- palette(nlevels(fac))[fac]

}

cex.points <- repeach(cex.points, nb)

}

points(pca$S[, c(xax, yax)], pch = pch.points, col = col.points,

cex = cex.points)

}

if (labels) {

s.label(pca$S, xax = xax, yax = yax, clabel = clabel,

cpoint = 0, boxes = boxes, add.plot = TRUE)

}

if (ellipses & !is.null(fac)) {

s.class(pca$S, xax = xax, yax = yax, fac = fac, clabel = clabel,

cpoint = 0, add.plot = TRUE, cstar = 0, col = NA,

cellipse = 0, axesell = FALSE)

}

if (eigen) {

par(mar = op)

add.scatter.eig(pca$Eval, nf = pca$sig.PCs, xax = xax,

yax = yax, eigen.ratio, posi = "bottomright")

}

if (draw.tree) {

tree <- pca$phy

X <- pca$S

if (class(tree) != "phylo")

stop("tree object must be of class 'phylo.'")

if (nrow(X) != length(tree$tip))

stop("X must contain the same number of rows as species in tree.")

if (is.null(rownames(X))) {

warning("X is missing row names; assuming order of tip labels.")

rownames(X) <- tree$tip.label

}

X <- X[, c(xax, yax)]

A <- apply(X, 2, fastAnc, tree = tree)

con = list(col.edge = setNames(rep("black", nrow(tree$edge)),

as.character(tree$edge[, 2])), col.node = setNames(rep("black",

max(tree$edge)), as.character(1:max(tree$edge))))

con[(namc <- names(control))] <- control

if (!is.null(tree$maps))

colors <- setNames(palette()[1:ncol(tree$mapped.edge)],

sort(colnames(tree$mapped.edge)))

lwd <- 1

aa <- setNames(c(X[tree$tip.label, 1], A[, 1]), c(1:length(tree$tip.label),

rownames(A)))

bb <- setNames(c(X[tree$tip.label, 2], A[, 2]), c(1:length(tree$tip.label),

rownames(A)))

XX <- matrix(aa[as.character(tree$edge)], nrow(tree$edge),

2)

YY <- matrix(bb[as.character(tree$edge)], nrow(tree$edge),

2)

points(x = A[1, 1], y = A[1, 2], pch = pch.points, col = col.points,

cex = cex.points)

if (is.null(tree$maps)) {

for (i in 1:nrow(XX)) lines(XX[i, ], YY[i, ], col = con$col.edge[as.character(tree$edge[i,

2])], lwd = lwd)

}

else {

for (i in 1:nrow(XX)) {

xx <- tree$maps[[i]]/sum(tree$maps[[i]]) * (XX[i,

2] - XX[i, 1])

yy <- tree$maps[[i]]/sum(tree$maps[[i]]) * (YY[i,

2] - YY[i, 1])

cc <- names(tree$maps[[i]])

x <- XX[i, 1]

y <- YY[i, 1]

for (j in 1:length(xx)) {

lines(c(x, x + xx[j]), c(y, y + yy[j]), col = colors[cc[j]],

lwd = lwd)

x <- x + xx[j]

y <- y + yy[j]

}

}

}

points(c(XX[i, 1], XX[tree$edge[, 2] > length(tree$tip.label),

2]), c(YY[i, 1], YY[tree$edge[, 2] > length(tree$tip.label),

2]), pch = 16, cex = 1)

points(XX[tree$edge[, 2] <= length(tree$tip.label), 2],

YY[tree$edge[, 2] <= length(tree$tip.label), 2],

pch = pch.points, cex = cex.points)

zz <- sapply(1:length(tree$tip.label), function(x, y) which(x ==

y), y = tree$edge[, 2])

if (tree.lab)

textxy(XX[zz, 2], YY[zz, 2], labs = tree$tip.label,

cx = 0.5)

}

par(mar = op)

}

####

# phylo.pca.contrib generates a plot illustrating the shape change along a given PC axis

####

phylo.pca.contrib <-

function (pca, PC.r = 1:pca$sig.PCs, sd = 2, cols = rep(NA, 3),

borders = c("#000080", "#000000", "#EE0000"), lwd = 1, nb.pts = 300,

plot = TRUE, legend = TRUE)

{

if ((length(PC.r) > pca$sig.PCs) | (max(PC.r) > pca$sig.PCs)) {

stop("The PC.r must correspond to PC axes present in the phylo pca object")

}

res <- list()

for (i in seq(along = PC.r)) {

pos.i <- sd * sd(pca$S[, PC.r[i]])

shp.i <- pca2shp.new(pos = matrix(c(-pos.i, 0, pos.i), nrow = 3),

rot = as.matrix(pca$Evec.full[, PC.r[i]]), mean.shp = pca$mean.shp,

method = "efourier", trans = FALSE, nb.pts = 300)

shp.i <- a2l(shp.i)

names(shp.i) <- paste0(rep(paste0("PC", PC.r[i]), 3),

c("-", "m", "+"))

res <- append(res, shp.i)

}

if (plot) {

op <- par(no.readonly = TRUE)

on.exit(par(op))

par(mar = c(1, 2, 1, 1), xpd = NA)

n <- length(PC.r)

pos <- cbind(1:n, matrix((n + 1):(4 * n), nrow = n, ncol = 3,

byrow = TRUE))

plot(NA, asp = 1, xlim = c(0, 4), ylim = c(0, n), xaxs = "i",

yaxs = "i", frame = FALSE, ann = FALSE, axes = FALSE)

res.t <- lapply(res, coo.template, size = 0.9)

for (i in 1:n) {

coo.draw(coo.trans(res.t[[(i - 1) + 1]], 0.5, n -

((i - 1) + 0.5)), col = cols[1], border = borders[1],

lwd = lwd, points = FALSE, first.point = FALSE)

coo.draw(coo.trans(res.t[[(i - 1) + 2]], 0.5, n -

((i - 1) + 0.5)), col = cols[2], border = borders[2],

lwd = lwd, points = FALSE, first.point = FALSE)

coo.draw(coo.trans(res.t[[(i - 1) + 3]], 0.5, n -

((i - 1) + 0.5)), col = cols[3], border = borders[3],

lwd = lwd, points = FALSE, first.point = FALSE)

}

for (i in 1:(n * 3)) {

pos.x <- rep(0:2 + 1.5, times = n)

pos.y <- rep((n - 1):0 * 1 + 0.5, each = 3)

coo.draw(coo.trans(res.t[[i]], pos.x[i], pos.y[i]),

col = cols[((i - 1)%%3) + 1], border = borders[((i -

1)%%3) + 1], lwd = lwd, points = FALSE, first.point = FALSE)

}

if (legend) {

text(1.5, n, labels = paste("-", sd, "s.d.", sep = ""),

adj = 0.5)

text(2.5, n, labels = "Mean", adj = 0.5)

text(3.5, n, labels = paste("+", sd, "s.d.", sep = ""),

adj = 0.5)

text(0, (n:1) - 0.5, labels = paste("PC", PC.r),

adj = 1)

}

}

invisible(res)

}

**Step-By-Step Guide to R Script Usage:**

**Step 1: Open R:**

Start R and execute the script above to load all of the required functions. Be sure to load the required libraries (Momocs, ape, geiger, phytools, and calibrate)

**Step 2: Input and Prepare data:**

efa.phyl.pca.R can utilize EFA data from two different sources: either a Coe object created with the Momocs R package [1] or a .nef file of normalized elliptical Fourier descriptors produced by the SHAPE software package [2]. If a .nef file is to be used, it must be imported and converted to a Coe object as follows. With normalized elliptical Fourier data, the coefficients A1, B1, and C1 are constants and should be deleted before analysis using the neftrimmer function.

coeA<-NEF2COE("your_file_name_here.nef")

data<-coeA@coe

dataA<-neftrimmer(data)

**Step 3: Import Phylogenetic Tree:**

A phylogenetic tree should then be imported as a phy object. One way to do this to import a topology from a Nexus file as shown below. The topology must have tip labels and branch lengths.

tree1 <- read.nexus("your_file_name.nex")

**Step 4: Estimate phylogenetic signal:**

efa.phyl.pca.R contains a function for estimating phylogenetic signal in EFA shape data using the consistency index [6]. Note that this function can take a very long time to execute, especially on older computers.

efa.phylo.signal(tree1,dataA,nharm=8,iter=1000)

**Step 5: Calculate Phylogenetic PCA**

Phylogenetic PCA of elliptical Fourier shape data can now be calculated. The number of harmonics to retain must be specified. This should be calculated using the Fourier power equation [7,8]. In this example, 8 harmonics will be retained.

PCA<-efa.phyl.pca(tree1,dataA,nharm=8)

PCA will be a list containing the following objects:

| mean.shp | matrix of the NEF descriptors of the mean shape |
| --- | --- |
| num.harms | the number of harmonics retained |
| Eval | the eigenvalues |
| Evec | eigenvectors, without values for A1, B1, and C1, the harmonic coefficients that were removed for the PCA |
| S | principal components scores |
| L | loadings on each axis, without values for A1, B1, and C1, the harmonic coefficients that were removed for the PCA |
| L.full | loadings on each axis WITH values for A1, B1, and C1. this can be used for plotting and subsequent analyses |
| Evec.full | Eigenvectors WITH values for A1, B1, and C1. this can be used for plotting and subsequent analyses |
| var.contrib | a vector showing the percent of total variance explained by each pricipal component axis |
| sig.PCs | the number of PC axes that explain 5 percent or greater of the cumulative variance |
| phy | the phylogenetic tree used for the analysis |

**Step 6: Plot Results:**

The phylo.efa.plot function, a derivation of the dudi.plot function of the Momocs package [1], is an extremely powerful and customizable tool for plotting the results of a phylogenetic PCA of EFA data in phylomorphospace

#phylomorphospace plots

#just points for tip taxa:

phylo.efa.plot(PCA2,shapes= FALSE,star=FALSE,ellipses=FALSE, pch.points=20)

#place taxon names centered over the appropriate points on the plot:

phylo.efa.plot(PCA2,shapes= FALSE,star=FALSE,ellipses=FALSE, labels =TRUE, pch.points=20)

#Inlude phylogenetic tree, ancestral taxa, and reconstructed ellipses on the plot:

phylo.efa.plot(PCA2,points=FALSE, shapes=TRUE,star=FALSE,ellipses=FALSE, draw.tree=TRUE)

#same as previous plot but with tip labels adjacent to data points

phylo.efa.plot(PCA2,points=FALSE, shapes=TRUE,star=FALSE,ellipses=FALSE, draw.tree=TRUE, tree.lab=TRUE)

**References**

1. Bonhomme V, Picq S, Gaucherel C, Claude J (2013) Momocs: Outline Analysis Using R. Journal of Statistical Software: InReview.

2. Iwata H, Ukai Y (2002) SHAPE: a computer program package for quantitative evaluation of biological shapes based on elliptic Fourier descriptors. The Journal of Heredity 93: 384–385.

3. Revell LJ (2009) Size-Correction and Principal Components for Interspecific Comparative Studies. Evolution 63: 3258–3268.

4. Adams DC, Otárola-Castillo E (2013) geomorph: an R package for the collection and analysis of geometric morphometric shape data. Methods in Ecology and Evolution 4: 393–399.

5. Revell LJ (2011) phytools: an R package for phylogenetic comparative biology (and other things). Methods in Ecology and Evolution 3: 217–223.

6. Klingenberg CP, Gidaszewski NA (2010) Testing and Quantifying Phylogenetic Signals and Homoplasy in Morphometric Data. Systematic Biology 59: 245–261.

7. Crampton JS (1995) Elliptic Fourier Shape-Analysis of Fossil Bivalves - Some Practical Considerations. Lethaia 28: 179–186.

8. Claude J (2008) Morphometrics with R. New York: Springer. 316 p.
